# Right to health and encouragement of social participation in elementary education*

**DOI:** 10.17533/udea.iee.v41n1e08

**Published:** 2023-03-14

**Authors:** Maria Luiza dos Santos Barbosa, Carla Aparecida Arena Ventura, Marina Liberale, Raquel Helena Hernandez Fernandes

**Affiliations:** 1 Undergraduate student of the Bachelor's Degree and Degree Course in Nursing, Brazil. Ribeirão Preto School of Nursing (EERP), University of São Paulo (USP), Ribeirão Preto, São Paulo, Brazil Email: marialuizabarbosa@usp.br. Universidade de São Paulo Ribeirão Preto School of Nursing (EERP) University of São Paulo Ribeirão Preto São Paulo Brazil marialuizabarbosa@usp.br; 2 Full Professor of the Department of Psychiatric Nursing and Human Sciences (DEPCH). Ribeirão Preto School of Nursing (EERP), University of São Paulo (USP), Ribeirão Preto, São Paulo, Brazil Email: caaventu@eerp.usp.br Universidade de São Paulo Department of Psychiatric Nursing and Human Sciences (DEPCH) Ribeirão Preto School of Nursing (EERP) University of São Paulo Ribeirão Preto São Paulo Brazil caaventu@eerp.usp.br; 3 Master Nurse. Laboratory Specialist. Ribeirão Preto School of Nursing (EERP), University of São Paulo (USP), Ribeirão Preto, São Paulo, Brazil Email: liberale@eerp.usp.br Universidade de São Paulo Laboratory Specialist Ribeirão Preto School of Nursing (EERP) University of São Paulo Ribeirão Preto São Paulo Brazil liberale@eerp.usp.br; 4 Lawyer. Graduate student of the Psychiatric Nursing Program. Ribeirão Preto School of Nursing (EERP), University of São Paulo (USP), Ribeirão Preto, São Paulo, Brazil Email: raquelhhfernandes@usp.br Universidade Federal de São Paulo Ribeirão Preto School of Nursing (EERP) University of São Paulo (USP) Ribeirão Preto São Paulo Brazil raquelhhfernandes@usp.br

**Keywords:** health promotion, education, primary and secondary, simulation training, health councils, empowerment, adolescent., promoción de la salud, educación primaria y secundaria, entrenamiento simulado, consejos de salud, empoderamiento, adolescente, promoção da saúde, ensino fundamental e médio, treinamento por simulação, conselhos de saúde, empoderamento, adolescente

## Abstract

**Objective.:**

Develop and validate an ordinary meeting simulation template of the Municipal Health Council applied to students of cycle II of Elementary School.

**Methods.:**

Qualitative and descriptive research developed in two phases: construction of simulation scenario of ordinary meeting of the Municipal Health Council and validation by committee of experts who analyzed the representativeness and adequacy of the content. The scenario included the items: prebriefing, additional information about the case, scenario objectives, evaluation criteria (observers), scenario duration time, human and physical resources, instructions for the actors, context, references and debriefing. In order to be able to understand which items should be modified according to the evaluations of the experts, it was used as criterion that only items that had 80% or higher percentage of agreement between the experts for modification would be modified.

**Results.:**

There was agreement to modify the prebriefing in: additional information about the case (100%); learning objectives (88.8%); human and physical resources (88.8%); context (88.8%); and in the debriefing (88.8%). The prebriefing did not reach the level of agreement: evaluation criteria (66.6%), duration of the scenario (77.7%), instruction for authors (77.7%), references (77.7%), which were modified

**Conclusion.:**

With the template developed and then validated by the committee of experts, it will be possible to develop in the classroom content related to the right to health and social participation in the scope of elementary education, as well as encourage engagement in important bodies for the maintenance of democracy, justice and social equity.

## Resumen

**Objetivo.:**

Desarrollar y validar una plantilla de simulación de una reunión ordinaria del Consejo Municipal de Salud aplicada a estudiantes del ciclo II de Enseñanza Fundamental.

**Métodos.:**

Investigación cualitativa descriptiva desarrollada en dos fases: construcción de un escenario para simular una reunión ordinaria del Consejo Municipal de Salud y validación por un comité de expertos que analizó la representatividad y adecuación del contenido. El escenario incluyó los ítems: *prebriefing*, información adicional sobre el caso, objetivos del escenario, criterios de evaluación (observadores), tiempo de duración del escenario, recursos humanos y físicos, instrucciones para los actores, contexto, referencias y *debriefing*. Para validar el escenario, se definió un nivel de acuerdo entre los expertos de mínimo 80%.

**Resultados.:**

Hubo acuerdo para mejorar en el *prebriefing*: información adicional sobre el caso (100%); objetivos de aprendizaje (88,8%); recursos humanos y físicos (88.8%); contexto (88.8%); y en el *debriefing* (88.8%). En el *prebriefing* no alcanzó el nivel de acuerdo: criterios de evaluación (66,6%), tiempo del escenario (77.7%), instrucciones para los autores (77.7%), y las referencias (77.7%), los cuales fueron posteriormente modificados.

**Conclusión.:**

Con la plantilla desarrollada y posteriormente validada por expertos será posible realizar en el aula contenidos sobre el derecho a la salud y a la Participación Social en el ámbito de la educación básica, así como fomentar el compromiso en órganos importantes para el mantenimiento de la democracia, la justicia y la equidad social.

## Resumo

**Objetivo.:**

Desenvolver e validar um *template* de simulação de reunião ordinária do Conselho Municipal de Saúde aplicado em alunos do ciclo II do Ensino Fundamental.

**Método.:**

Pesquisa qualitativa e descritiva desenvolvida em duas fases: construção de cenário de simulação de reunião ordinária do Conselho Municipal de Saúde e validação por comitê de especialistas que analisaram a representatividade e adequação do conteúdo. O cenário contemplou os itens: *prebriefing*, informações adicionais sobre o caso, objetivos do cenário, critérios de avaliação (observadores), tempo de duração do cenário, recursos humanos e físicos, instruções para os atores, contexto, referências e *debriefing*. Para que fosse possível entender quais itens deveriam ser modificados de acordo com as avaliações dos especialistas, utilizou-se como critério que somente os itens que tivessem 80% ou porcentagem superior de concordância entre os especialistas para modificação é que seriam modificados

**Resultados.:**

Houve concordância para modificação em el prebriefing em: informações adicionais sobre o caso (100%); objetivos de aprendizagem (88.8%); recursos humanos e físicos (88.8%); contexto (88.8%); y em el *debriefing* (88.8%). Não atingiram o nível de concordância o *prebriefing:* critérios de avaliação (66.6%), tempo de duração do cenário (77.7%), instrução para autores (77.7%), referências (77.7%), os quais foram modificados.

**Conclusão.:**

Com o *template* desenvolvido e despois validado pelo comitê de especialistas será possível desenvolver em sala de aula conteúdos referentes ao direito à saúde e à Participação Social no âmbito da educação básica, bem como encorajar o engajamento em órgãos importantes para manutenção da democracia, justiça e equidade social.

## Introduction

The Brazilian National Health Promotion Policy (NHPP), published in 2006 by the Ministry of Health and revised in 2014, represents a political and ideological framework in the organization and implementation of actions within the Unified Health System (SUS). At its base, it presents important references as an expanded concept of health and the theoretical reference of health promotion. In the document, health promotion is understood “as a set of strategies and ways to produce health, at the individual and collective levels” and highlights the importance of broad participation and social control, as it has as its principle the strengthening of Social Participation (SP), autonomy, empowerment and teamwork. In addition, it understands the school as a territory and singular *locus* for the practice of health promotion.^1^ Another important political/ideological framework that strengthened and expanded health actions in the school environment within the scope of Primary Health Care was the Health at School Program ^(^^2^ (HSP), created in 2007. In it, the school space is recognized as privileged for the development of health promotion and health education practices, and, therefore, an environment conducive to the development of teaching strategies that stimulate SP, autonomy and empowerment. 

The SP refers to the actions that various social forces perform in order to influence the formulation, execution and evaluation in the public spheres in the social area.^3^ It calls on citizens to act together in defense of a common goal, in order to intervene in impasses that are present in the community, acting on a shared need.^4^ According to the World Health Organization,^5^ the SP has an intrinsic value to guarantee the autonomy of citizens and their right to be involved in decision-making actions that affect their health and disease process. In this way, it can make public representation more equitable, involving groups in vulnerable situations, ensuring legitimacy and combating conflicts of interest by those who say they represent the population.

The culture of the SP is one of the essential requirements for the effectiveness of social control in the daily life of the population. The Municipal Health Council (MHC) represents one of the channels of institutional participation used to enable social control, since, through this permanent and deliberative body, the organized society can appropriate the means and instruments of planning, inspection of public resources and analysis of health services.^6^ Within the SP, youth participation is fundamental for young people and adolescents to be promoters of social transformation, through active and autonomous participation in the planning, execution and evaluation of actions in the context of health, assisting in the effectiveness, resoluteness and social impact of public policies, not acting only as users of health services and programs.^7^

Since 2017, the authors of this article have developed a culture and outreach project linked to the Unified Scholarship Program (USP) of the University of São Paulo (USP) entitled “Leadership and Social Mobilization: stimulating the understanding of social participation in health in elementary public education students”, which was first developed in a municipal school and now in a state school, both in a city in the countryside of São Paulo. Based on this experience, it was possible to observe an impasse during the activities of the project: most of the students of Elementary Education did not know the MHC as a form of social control, besides manifesting little active participation in the community in which they live, with regard to the claim of their health rights. In the literature, it is possible to identify that, although community participation is one of the organizational principles of the SUS, there is still little adherence to the MHC by the popular strata for the claim of rights. This is due, in many cases, to the invisibility of the councils, precariousness in the communication process and scarcity of information, which includes information about its existence. In addition, there is also the discouragement to engagement and action in this body.^8^

Nursing can contribute to strengthening this scenario, acting within schools, in carrying out educational activities that go beyond the biomedical and curative model, especially because it is up to health managers to foster the creation in the Health Units of Local Health Councils (LHC). This council is formed, through elections, by a group of users, health workers and representatives of the Residents' Associations who participate in the monitoring of the activities of the Health Units and the care offered, discussing the problems, evaluating the quality and resoluteness of the actions developed and proposing solutions. Therefore, investing in education for youth protagonism will strengthen the very functioning of Health Units. Considering the importance of the participation of the population in the MHC, this article aims to develop and partially validate an ordinary meeting simulation template of the Municipal Health Council to be applied to students of cycle II of Elementary School.

## Methods

Type or design of the study. This was a qualitative and descriptive research, guided by the Revised Standards for Quality Improvement Reporting Excellence (SQUIRE 2.0) guide of the Equator Network. It contemplated two phases: the construction of the simulation scenario of an ordinary meeting of the MHC and the evaluation by a committee of experts who analyzed the representativeness and adequacy of the content. The survey was developed between August 2019 and August 2020. 

### Phase 1: Construction of the simulation scenario

The construction of the simulation scenario was based on research in the literature on simulation strategy and the experience of the authors of this article in the aforementioned culture and outreach project. In the stage of construction of the simulation scenario, five educational activities were developed with the students of Elementary School II of a state school in the city of Ribeirão Preto (SP), focusing on the identification of violations of health rights. The themes developed were: concept of health, rights and duties, forms of Social Mobilization, SP and MHC. The objectives of the meetings were: to reframe the concept of health; to discuss individual and collective rights and duties, to recognize the right to health and its violations; to identify community health problems; to know forms of intervention to solve problems; to propose forms of intervention and problem solving; to recognize the MHC as a form of intervention; to know the roles that involve the formation of a MHC; to exercise group work and communication skills; to develop critical thinking skills; and to reflect on the decision-making process. To achieve these objectives, we used as teaching strategies the conversation circle, small group work, playful activities, reading texts, making posters and presenting them in the classroom. The activities developed created conditions for students to identify in the community in which they live several violations of health rights. Among the violations identified, the discussions focused on the problem around the lack of medicines provided by the Unified Health System (SUS), enabling greater richness of details for the construction of the simulation scenario. For the construction of the simulation scenario, an adaptation of the template proposed by Fabri was used,^9^ contemplating ten items: prebriefing, additional information about the case, objectives of the scenario, evaluation criteria (observers), duration of the scenario, human and physical resources, instructions for the actors, context, references and debriefing.

### Phase 2: Evaluation by expert committee

Selection criteria. The following inclusion criteria were adopted: Elementary Education teachers who have been working for at least two years in Cycle II of Elementary School; specialists in simulation scenarios with experience for at least two years, according to data present in the curriculum of the Lattes Platform of the National Council for Scientific and Technological Development (CNPq); members of the MHC with at least two years in office, proven by the minutes of the meetings available on the platform of the Municipality. Elementary Education teachers, simulation specialists and MHC members who could not evaluate the simulation scenario within the timeframe stipulated by the research team were excluded from the study. 

Participants. In order for the scenario to be as faithful as possible to an ordinary meeting of a MHC, it was decided to validate it through a committee of experts. The number of experts was determined according to the recommendations of Polit and Beck,^10^ who propose that the committee be composed of six to ten experts. In this research, the committee was composed of 9 specialists, namely: 2 members of the MHC representing the category of health service users, 1 member of the MHC representing the category of health workers and 1 member of the MHC representing the category of managers and health service providers, in addition to 2 specialists in simulation scenarios and 3 teachers of elementary education (EE). 

Location or Scenario in which the data collection took place (city, state and country acronym). For the construction of the scenario, an experience of the Culture and Outreach project already mentioned was used. The teachers who participated in the data collection belong to the same institution and were chosen in view of the proximity to the problem listed in the neighborhood in which they live and with the team of this research. The counselors who were part of the data collection belong to the MHC of the same municipality.

Data collection. Elementary education teachers were contacted by telephone. The simulation scenario specialists were invited to participate in the research by email after a search on CNPq's Lattes Platform, using the descriptors “specialist” AND “simulation”. The contact with the board members took place at an Ordinary Meeting of the MHC, where the objective and method of the project were presented. Subsequently, after approval of the project by the directors at this meeting, those interested in being part of the validation of the simulation scenario were contacted by phone or email. All experts received, via e-mail, an invitation letter, the Informed Consent Form (ICF), the simulation scenario and an evaluation instrument. The experts evaluated the scenario separately according to their professional and personal attributions and experiences. 

Instruments used to collect information. . For the assessment instrument of the simulation scenario, an adaptation of the Likert-type scale was used. This instrument contemplated the elements of the scenario already mentioned, accompanied by three response items: adequate, partially adequate and not adequate, in addition to a space for suggestions and perceptions about the scenario. It was decided to use these three response items, since this configuration allows alternatives of sufficient responses, adjusting to small quantities of samples, in addition to requiring little response time from the evaluators. 

Treatment and data analysis After the feedback of the template evaluation instrument, a virtual meeting was held with the research team in order to discuss the evaluators' suggestions and which ones would be adopted according to their importance for the purpose of the template,. Thus, it was adopted that the items would be modified if there was agreement between the experts to modify the items in the percentage of 80% or higher^11^^-^^13^ and, for this, the calculation "number of participants who agreed /total number of participants X 100" was taken into account.^12^ Also, it should be noted that each item was evaluated individually, observing the suggestions for modification and applying the calculation above, so that they obtained its own percentage.

Ethical aspects. The construction and validation of the simulation scenario was initiated after approval by the Ethics and Research Committee of the Ribeirão Preto School of Nursing of the University of São Paulo under number 14011519.7.0000.5393, respecting Resolution 580/12 on ethical requirements for research with human beings. It is important to mention that the Educational Institution granted authorization to carry out this research. 

Methodological aspects. The simulation scenario was elaborated by surveying the contents related to the theme in the literature, scientific articles and the experience of the research team in the municipality's MHC and in the culture and outreach project already mentioned. The pre-simulation, prebriefing, instructions for the authors and references were prepared so that the students could develop the knowledge, skills and habits necessary to carry out the simulation and decision making. The context and additional information about the case were structured through a health problem identified by the students: lack of medicines in the SUS. In this sense, we sought to organize these elements with everyday situations, incorporating in the simulation the experiences of students. The objectives of the scenario were constructed in order to be understandable to students, taking into account knowledge, skills and capacities that are essential to be assimilated both for the realization of the simulation scenario, as for future situations, in school and in practical life. 

With regard to the development of the elements of assessment criteria and *debriefing*, consideration was given to the assessment of learning objectives. The duration of the scenario and the necessary resources (human, physical and material) were developed based on the experience that the team of this research had previously with EE students.

## Results

The expert committee was composed of 55.6% female and 44.4% male. Regarding the agreement index, there was agreement to modify five items, namely: additional information about the case; learning objectives; human and physical resources; context and debriefing. On the other hand, the following items were not reached the level of agreement for modification: prebriefing and evaluation criteria, in which the consensus among experts was 66.6%; and in the items of duration of the scenario, instruction for authors, references, in which the consensus was 77.7% (Table 1).

**Table 1:** Index of agreement among experts

Items	Adequate	Partially adequate	Inadequate	Concordance Index (%)
Prebriefing	6	3	-	66.66
Additional information about the case	9	-	-	100
Learning Objectives	8	1	-	88.88
Evaluation Criteria	6	3	-	66.66
Scenario Duration Time	7	2	-	77.77
Human and Physical Resources	8	1	-	88.88
Instruction for authors	7	2	-	77.77
Context	8	1	-	88.88
References	7	1	1	77.77
Debriefing	8	1	-	88.88

According to the recommendation of the experts, there were changes in the duration of the simulation, totaling one hour and fifty minutes, being: twenty minutes for prebriefing, fifty minutes for the simulation scenario and forty minutes for debriefing. Changes in the prebriefing item were made so that they could give pertinent information to subsidize and guide students during their performance in the simulation scenario. In this sense, details were included about the organization and dynamics of an ordinary meeting of the MHC such as speaking time, prior registration and possibility of re-enrollment of the directors for the pronouncement, detailing, in this case, the criteria necessary for acceptance by the President of the MHC. 

As a result of these modifications, the attributions of the President of the MHC were improved, in the item instructions for authors, with regard to the function of overseeing the order of registration in granting the floor to board members It is important to note that since the purpose of the template is to be faithful to reality, it was decided to accept the evaluator's suggestion, although the aforementioned changes may vary from municipality to municipality, according to the Internal Regulations of the MHC. Regarding the evaluation criteria, the changes made focused on specifying what is expected of students in an objective way, highlighting the attitudes and concepts that demonstrate that such objectives were achieved. The references were adapted, including two documents: the Federal Constitution of 1988, which guaranteed the participation of society in the management of public policies and the Internal Regulations of a MHC in the countryside of the State of São Paulo, in order to bring students closer to the functioning and function of this deliberative body. 

In general, the experts suggested adapting the language of the template in order to facilitate the understanding of the EE student in the simulation scenario, making the template clearer and objective. Therefore, the results of the validation by the experts were essential to make relevant changes in this simulation scenario. These modifications resulted in the final version of the simulation scenario of a MHC meeting, which is presented below in Table 2.

**Table 2:** Template for construction of scenarios for simulation of an ordinary meeting of the Municipal Health Council

Scenario title: The lack of supply of medicines in pharmacies of basic health units and the Municipal Health Council		
Target audience: Elementary School students II of the public network		
Number of Participants: 30 - 40 students		
Pre-simulation		
Develop with the students the following themes: concept of health; identification of health problems; forms of social participation, Municipal Health Council and the Internal Regulations in the Municipal Health Council. In this *template*, after the development of the themes mentioned above, the following health need was listed: Supply of medication by the Unified Health System. We realized that, if there is a long period between the themes and the simulation, it is important to allocate a meeting as a way to resume the contents.		
The pre-simulation should provide theoretical and practical elements in relation to the health need listed and aims to work three meetings lasting 1 hour and 20 minutes for the themes:		
Access to population information and access to medicines by the Unified Health System		
Forms of obtaining the medicine by the Unified Health System		
National and Municipal List of Essential Medicines		
Causes and consequences of the lack of supply of medicines by the Unified Health System		
Prebriefing		
Participants will be informed that:		
The scenario is an ordinary meeting of the Municipal Health Council		
The duration of the simulation will be approximately 50 minutes		
The agenda of the meeting is: lack of supply of medicines by the Unified Health System		
Those who wish to have the right to speak must register with the President of the Council in advance. Each registered board member will have a maximum of 3 (three) minutes for his intervention, and re-registration will only be accepted (by the Chairman of the Board) if the time allocated to the theme "Lack of medicines by the Unified Health System" so allows, with the option of new registrations on re-registrations		
The objective of the scenario is to:		
Analyze the health problem		
Identify the risks to the health of the population		
Propose a resolution/referral to the problem		
Demonstrate group leadership skills		
The acting roles in the scenario are: Chairman of the Board, Representatives of Users, Representatives of Health Professionals, Government Representative and Observers		
The simulation will be finalized when the Chairman of the Board closes the meeting		
Context of the meeting: “At a meeting of the Popular Health Movement in the neighborhood of Vila Virginia, it was pointed out that the medicines available at the neighborhood health unit were missing or with insufficient deliveries, especially medicines for hypertension and diabetes. As one of the results, most users have to move, often on foot, to another neighborhood to try to get the medicine for the continuation of treatments. In addition, at this meeting, it was pointed out that most of the residents of the neighborhood do not have information on which medicines are available through the Unified Health System network and, due to this, they allocate a large part of their income to the purchase of medicines that they could get for free. The members of the Popular Health Movement, recognizing the importance of improving this situation, decided to take this situation to the Local Health Council. In turn, the Local Health Council took this problem to be discussed at an ordinary meeting of the Municipal Health Council”.		
Additional information about the case		
The case is put on the agenda by the representatives of the users		
There has been a shortage of medicines for hypertension and diabetes in the Unified Health System network for more than 40 days		
Due to the lack of medicines for hypertension and diabetes, the neighborhood health unit is with more demanding and complication from these diseases.		
The distance between the neighborhoods is 30 to 40 minutes on foot		
Most residents do not own a car.		
Scenario Objectives:		
Main objective		
Propose an intervention measure for the scenario problem: “lack of supply of medicine by the Unified Health System”.		
Specific objectives		
Exercise leadership skills and social mobilization		
Identify individual and/or collective health problems		
Know forms of intervention to solve health problems		
Know the Municipal Health Council as a form of intervention		
Know the roles that involve the formation of a Municipal Health Council		
Exercise group work skills		
Strengthen and integrate the knowledge acquired in the pre-simulation phase, linking theoretical knowledge to practice.		
Assessment criteria		
Assessed items	**Assessment *Grade: 0 to 5 or N- Not evaluated***	
Recognized the theme of the simulation scenario as an individual and collective health problem		
They worked in groups through discussions.		
Demonstrated leadership skills		
Proposed form of intervention for the problem		
They recognized the Municipal Health Council as a means of social control and a space for discussion of health-related aspects		
Analyzed the problem in question critically relating the impacts of the problem on people's lives collectively		
Showed respect to other participants		
They established priorities regarding the needs of the population in relation to the lack of supply of medicines in the Unified Health System		
Recognized the problem as a public health issue		
Identified the risks to the health of the population		
Scenario Duration Time		
The simulation will take about 1 hour and 50 minutes, and will be destined:		
Prebriefing:20min		
Simulation: 50min		
Debriefing: 40min		
Human and Physical Resources		
Human Resources	Physical Resources	Material
Elementary students	Environment	Sulfite papers
Facilitators (research team)	Classroom	Tables
Elementary school teachers		Chairs
		Pens
		Agenda of the meeting (information about the cases and problem in question)
		Camera and recorders
Instructions for actors		
For the execution of the scenario, the students will be divided into 5 roles, among them: chairman of the board, representative of users, representatives of health professionals, representative of the government and observers. It is essential to emphasize that the division of the roles played by the students will be carried out consensually by the students themselves.		
Thus, the roles that make up the scenario are:		
Chairman of the Board: 1 participant. Responsible for opening the meeting, finalizing the meeting, as well as conducting it in relation to organizing the turn of each of the members of the scenario (representatives of the users, representatives of the health professionals and representatives of the government) to position themselves on the agenda of the meeting (register the counselors), in addition to conducting the time of the meeting and the time of the vote, among other factors. In addition, it is up to the President of the Council to ensure the order of registration in the concession of the floor and the non-concession of the floor when the registration is finalized.		
Representatives of Users: 50% of participants in the simulation scenario (except observers). Group composed of residents of a certain region, responsible for charging public agencies about the needs of the neighborhood, in the search for improvements in the distribution of the medicine in the pharmacy of the primary care health unit in the neighborhood. Thus, it seeks improvements for that place, representing the interests of users of the health service offered by the SUS, bringing to the meeting the needs of the community. In the proposed scenario, there are representatives of the *Popular Health Movement of the Vila Virginia neighborhood in this group.*		
Healthcare Professionals Representatives: 25% of participants in the simulation scenario (except observers). They represent professionals as physical therapists, nurses, physicians, among other health professionals. In the simulation scenario, it makes up the group of *Representatives of General Union Entities*.		
Government Representative (City Hall of Ribeirão Preto): 25% of the participants in the simulation scenario (except observers). It is responsible for guiding, coordinating and executing activities related to the structure of the city and the environment. In this case, the representative of the government will be *the Health Department of the city of Ribeirão Preto*, since it is a meeting of the Municipal Health Council.		
Observers: The observers will be students who will not be participating as one of the representatives of the aforementioned Municipal Health Council. They will be responsible for observing the entire scene, listing the positives and negatives of the performances, the simulation scenario and what they would do differently. During the simulation scenario, observers will be divided into four (4) small subgroups to observe the performance of each representative group of the Municipal Health Council. At this point, you will be tasked with completing a document with the skills and characteristics observed during the simulation.		
Context		
“At a meeting of the Popular Health Movement in the neighborhood of Vila Virginia, it was pointed out that the medicines available at the neighborhood health unit were missing or with insufficient deliveries, especially medicines for hypertension and diabetes. As one of the results, most users have to move, often on foot, to another neighborhood to try to get the medicine for the continuation of treatments. In addition, at this meeting, it was pointed out that most of the residents of the neighborhood do not have information on which medicines are made available by the Unified Health System network and, due to this, they allocate a large part of their income to the purchase of medicines that they could obtain gratuitously. The members of the Popular Health Movement, recognizing the importance of improving this situation, decided to take this problem to the Local Health Council. In turn, the Local Health Council took this matter to be discussed at an ordinary meeting of the Municipal Health Council”.		
“At a meeting of the Popular Health Movement in the neighborhood of Vila Virginia, it was pointed out that the medicines available at the neighborhood health unit were missing or with insufficient deliveries, especially medicines for hypertension and diabetes. As one of the results, most users have to move, often on foot, to another neighborhood to try to get the medicine for the continuation of treatments. In addition, at this meeting, it was pointed out that most of the residents of the neighborhood do not have information on which medicines are made available by the Unified Health System network and, due to this, they allocate a large part of their income to the purchase of medicines that they could obtain gratuitously. The members of the Popular Health Movement, recognizing the importance of improving this situation, decided to take this problem to the Local Health Council. In turn, the Local Health Council took this matter to be discussed at an ordinary meeting of the Municipal Health Council”.		
References - Suggestions		
ACURCIO, F. A.; BRANDÃO, C. M. R.; FALEIROS, D. R.; Jr, A. A. G.; CHERCHIGLIA, M. L.; ANDRADE, E. G. Judicialization of access to medicines in the State of Minas Gerais, Brazil. **Rev. Saúde Pública**, São Paulo, v. 45, n. 3, p. 590-598, 2011.		
BOING, A. C.; BERTOLDI, A. D.; BOING, A. F.; BASTOS, J. L.; PERES, K. G. Access to medicines in the public sector: analysis of users of the Unified Health System in Brazil. **Cad. Saúde Pública**, Rio de Janeiro, v. 29, n. 4, p. 691-701, Apr. 2013.		
BRASIL. **National List of Essential Medicines 2020**. Brasília: Ministério da Saúde; 2019.		
BRASIL. Constituição (1988). **Constituição da República Federativa do Brasil**. Brasília, DF: Senado Federal, 2018.		
LEITE, R. A. F.; BRITO, E. S.; SILVA, L. M. C.; PALHA, P. F.; VENTURA C. A. A. Access to information in health and comprehensive care: perception of users of a public service. **Interface**, Botucatu, v. 18, n. 51, p. 661-671, 2014.		
PORTELA, A. S.; LEAL, A. A. F.; WEBER, R. P. B.; SIMÕES, M. O. S.; MEDEIROS, A. C. D. Policies of medicines: trajectory and challenges. **Revista de Ciências Farmacêuticas Básica e Aplicada**, São Carlos, v. 31, n. 1, p. 09-14, 2010.		
CITY HALL OF RIBEIRÃO PRETO. Remune: Municipal List of Essential Medicines. Ribeirão Preto. Available from: <https://www.ribeiraopreto.sp.gov.br/ssaude/saudepessoal/farmacia/i16remume.php>. Accessed May 18, 2020.		
REIS, AMM; PERINI, E. Medicine shortages: determinants, consequences and management. **Ciência & Saúde Coletiva**, Rio de Janeiro, v. 13, p. 603-310, 2008.		
RIBEIRÃO PRETO Law No. 221, of August 1, 2018. Approval on the internal regulations of the Municipal Council of Ribeirão Preto. **Official Gazette of the Municipality of Ribeirão Preto**. 01 Aug. 2018.		
Debriefing		
The following guiding questions will be carried out for the simulation participants (students):		
Debriefing Phase		
	GUIDING QUESTION	
Descriptive Phase	Questions for all scenario participants:	
	How did you feel about attending a City Health Council meeting?	
	What were the feelings, emotions and perceptions expressed during the meeting of the Municipal Health Council?	
	How important was the meeting of the Municipal Health Council for the development of self-confidence?	
Analytical Phase	Questions for scenario participants who served as board president, user representative, health worker representatives, government representative.	
	At the time of the meeting, what would you do differently?	
	Considering the aspects listed in the objectives in the first phase of the simulation, what objectives do you think you were able to achieve?	
	What were the objectives that were not achieved?	
	How do you consider your performance in group work?	
	What were the difficulties experienced in the case presented?	
	What were the difficulties/facilities presented in the process of promoting an intervention in the case of the meeting?	
	Do you have the knowledge and skills to achieve your objectives?	
	Questions directed to scenario observers:	
	What aspects were observed during the meeting of the Municipal Health Council?	
	What are the comments made on the proposed intervention by the members? Would you do anything differently?	
Application Phase	Question directed to all students:	
	What would you change in the roles that make up the meeting of the Municipal Health Council?	
	What would you change in the case presented?	
	What changes do you propose in the physical space of the scenario?	
	What can you take from the experience of this simulation to practice in the community in which you live?	
Notes:		

Source: Adapted from Fabri RP. Construction of practical script for simulated activity [dissertation]. Ribeirão Preto School of Nursing: University of São Paulo; 2015

## Discussion

Pre-simulation was important to understand the needs that students identify as being paramount in their communities, thus enabling a participatory space in the creation of the scenario and stimulating the development of critical and reflective thinking. One of the factors for the construction of knowledge is the apprehension of reality and problematization is one of the tools to enable the transformation of the context in which the students are inserted.^14^ From this, the objectives of the scenario were considered, thinking that they should be clear and understandable.^15^ Thus, the perspective is that the objectives can stimulate the SP, propose challenges, identify problems and issues, propose solutions and contribute to the decision-making process.

In addition, pre-simulation, prebriefing, instructions for authors and references were articulated by the research team so that they could establish a minimum standard of knowledge,^16^ skills and habits that students need to have contact to perform the simulation^17^ and that they give subsidies to guide students in taking positions and decisions regarding the theme worked in the simulation scenario. The evaluation criteria elements and the debriefing were built in order to provide self-understanding, both of the students and the research team, of the level and conditions in which the participants are regarding the understanding of the entire activity, since the evaluation criteria and the debriefing also aim to carry out a "diagnosis" of the activity developed, in order to direct the subsequent acts of the activity. In addition, these elements were elaborated with the function of motivating human development, given that, through the recognition of the breadth and limit of where it is, it proves to be a motivation to continue.^17^

It is important to mention that the composition of the committee of experts with participants from the areas related to the template proposal could provide the views of educators, simulation specialists and MHC counselors and thus ensure that learning objectives were achieved and the simulated experience more accurately portrayed reality.^18^According to authors references in education,^19^ the importance of creating a space for dialogue, in the construction of bridges and in which there is greater focus on the performance of students, enabling students to assume the role of protagonists in the teaching-learning process, an aspect present in the simulation process, stands out. As a result, it is expected the deepening of ideas and problems, given that simulation tends to develop critical thinking skills, problem solving and the decision-making process,^20^ contributing to the training of leaders who work in SP scenarios. Simulation is an important tool to privilege learning and student satisfaction, by training the skills mentioned and developing essential reflections on action in a context similar to practice. The simulation teaching method allows learning with and in experience (practical dimension), in addition to enabling the understanding of the importance of peer learning,^14^^,^^21^ leadership in mobilizing people, as well as critical reflection of the reality in which it is inserted.^14^

## Conclusion

Taking into account the objective of this research, the purpose of building a simulation *template* of an ordinary meeting of a MHC within the scope of Elementary Education was achieved. We developed a replicable simulation *template*, however, we understand that it is important to continue the validation with the application in the target audience. Thus, for future studies, it is essential to apply this simulation scenario as a way to evaluate its results in practice, with regard to the recognition of participants in the simulation scenario as an individual and collective health problem, and the MHC as a form of social control and a space for discussion of aspects related to health. 

It is expected that the template developed can be applied as a pedagogical strategy by teachers of Elementary Education, by health professionals of Basic Units, and by other researchers for the development of leadership and SP skills in health, adapting it according to the social and cultural differences of the different territories. Still in this sense, nurses can be a replicator of this template, since they are team leaders within hospitals, institutions and health organizations. Thus, the template can be an inspiration tool for discussing problems that permeate these places, from fostering searches for solutions involving other social agents.

Another limitation of the study is the fact that one of the validation stages was carried out in only one state school in the city of the countryside of the state of São Paulo and, therefore, addressing a specific social and cultural reality. It is worth considering that the counselors who participated in the validation of the *template* were from only one city in the countryside of São Paulo, and the *template* may have changes according to the region and the internal regulations of the MHC of the municipality in which it is applied.
